# Dual-Receptor Recognition, Lysis Inhibition, Endolysin Release, and Reaction–Diffusion as Alternative Explanations. Comment on Rojero et al. Bypassing Evolution of Bacterial Resistance to Phages: The Example of Hyper-Aggressive Phage 0524phi7-1. *Int. J. Mol. Sci.* 2025, *26*, 2914

**DOI:** 10.3390/ijms262311368

**Published:** 2025-11-25

**Authors:** Stephen T. Abedon

**Affiliations:** Department of Microbiology, The Ohio State University, Mansfield, OH 44906, USA; abedon.1@osu.edu

**Keywords:** dual-receptor generalist, endolysin, lysin, lysis inhibition, lysis-inhibition collapse, reaction-diffusion, star mutants

## Abstract

Presented here are additional explanations for five key points offered by Rojero et al. in their 2025 publication in this journal, regarding characteristics of hyper-aggressive phage 0524phi7-1. These are (i) that the “bypassing of the evolution of host resistance” has been seen in other phages, especially dual-receptor generalist phages; (ii) that the “clearing of semi-turbid plaques” could be due to a phenomenon known as lysis inhibition collapse, (iii) that the “formation of satellite plaques” is reminiscent of the morphology of plaques generated by phage T4 star mutants, (iv) that “multi-day plaque enlargement” has been seen in other phages such as phage T7 but may also be explained by other phenomena including endolysin release, (v) that suggestions of phage “swimming” could be explained by virion diffusion within empty volumes found within maturing bacterial lawns. In particular, phage plaques that display lysis inhibition can influence the surrounding bacterial lawn well beyond their visible region. This presumably occurs via a reaction–diffusion mechanism whose leading edge of virion diffusion fails to display lysis inhibition, but which leaves in its wake lysis-inhibited bacterial infections that may not lyse in a timely manner. Phage-infected bacteria thus may be found well beyond a plaque’s visible boundaries, along with diffusing endolysin.

## 1. Introduction

I have read with interest the article by Rojero et al. [1], “Bypassing Evolution of Bacterial Resistance to Phages: The Example of Hyper-Aggressive Phage 0524phi7-1”. The authors describe four characteristics of what they term a “hyper-aggressive phage”. I wish to address each of these four characteristics (Section 2, Section 3, Section 4 and Section 5), especially as illustrated in that article. This is to provide additional context from the existing literature by myself and others. Additionally, I provide an alternative mechanism to Rojero et al.’s proposed phage “swimming” (Section 6) that is based instead on the known ability of phage virions to diffuse within agar.

I emphasize that the *Bacillus thuringiensis* host of phage 0524phi7-1 is Gram-positive and that phage 0524phi7-1 potentially releases functional endolysin (lysin) into media upon the completion of phage lytic cycles. I also emphasize the potential for phage 0524phi7-1, as a myovirus, to display the classically known phage lysis inhibition phenotype.

## 2. “The Bypassing of the Evolution of Host Resistance”

I agree that this is a relevant phage characteristic, particularly for phage therapy applications. However, phages with similar characteristics have been previously described. Borin et al. [2] in particular characterized phages with comparable properties as “dual-receptor generalists”. Additionally, numerous phages have been independently shown to recognize multiple receptors, including receptors found on the same individual bacterial cells [3,4,5].

## 3. “The Clearing of Semi-Turbid Plaques”

**Figure 1:**
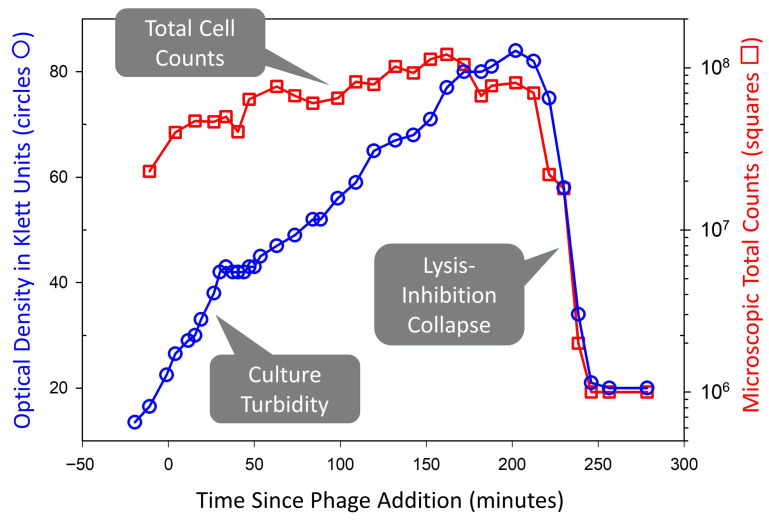
Lysis inhibition and lysis inhibition collapse. Shown is phage T4 replicating on *Escherichia coli* CR63. This was monitored simultaneously in terms of both optical density and total cell counts (the latter determined using a Petroff-Hausser counting chamber). Cultures were initiated with a phage multiplicity of somewhat less than one, allowing exponential bacterial growth until phage titers caught up with bacterial densities. This was then followed by lysis of a portion of the now mostly phage-infected bacterial population (flattening of the optical density curve around 50 min). Shortly after, lysis inhibition was initiated in the rest of the phage-infected bacterial population. The rise in optical density after 50 min is due to increasing bacterial size [15,16] rather than substantial division of phage-infected cells. The rapidity of the lysis inhibition collapse starting after 200 min is associated with a positive feedback mechanism that may involve the phage T4 lysis-from-without phenomenon [10]—lysing lysis-inhibited bacteria supply multiple virions that secondarily adsorb still-intact phage-infected bacteria, motivating their lysis [10]. This collapse still occurs, albeit more slowly, even if further phage secondary adsorption of lysis-inhibited bacteria is blocked [11]. This figure is based on the sixth figure presented in Abedon [10] and is reproduced under a Creative Commons Attribution (CC BY) license.

Although the specific cause of the plaque turbidity observed by Rojero et al. [1] remains uncertain, one possible explanation involves the lysis inhibition phenomenon seen in some obligately lytic phages [6,7,8,9,10]. Lysis-inhibited, phage-infected bacteria undergo lysis after extended infection periods, a process termed “lysis-inhibition collapse” [11,12,13,14] (Figure 1). This delayed “collapse” phenomenon could account for the clearing that was observed by Rojero et al. It should be noted, however, that lysis inhibition does not necessarily indicate that a phage is hyper-aggressive, but instead that it is a phage that can be conditionally slow to lyse its host.

Additionally, the semi-turbidity of phage 0524phi7-1 plaques might result from lawn microcolonies requiring additional time to become fully infected and then fully lysed, particularly under conditions of lysis inhibition [17]. That is a possibility that may be supported by the successful isolation of phage-uninfected bacteria following streaking from “semi-turbid 18 h incubation”, as reported by the authors [1]. That is, bacterial colonies resulting after plating perhaps originated as uninfected bacteria previously trapped within still partially intact lawn microcolonies. Note that the two possibilities presented in this section are not mutually exclusive—lysis inhibition collapse and microcolonies that are not fully phage-infected. A further consideration of the subsequent lysis of these bacteria and thus the clearing of these semi-turbid regions of plaques is provided in Section 5.4.

## 4. “The Formation of Satellite Plaques”

**Figure 2:**
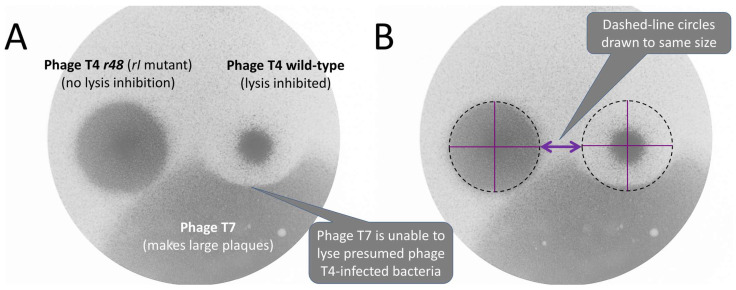
The area of influence of lysis-inhibited plaques extends beyond their visible boundaries. Panel (**A**): Plaques of wild-type phage T4 which displays lysis inhibition and the phage T4 *r48* rapid lysis mutant which does not display lysis inhibition, both colliding with phage T7. Phage T7, was used because it produces very large plaques [22,23], which here extended well beyond the presented field of view. Panel (**B**): The circle that is centered on the wild-type phage T4 plaque indicates that its area of influence appears to extend substantially beyond its visibly cleared center, as revealed by its interference with the phage T7 plaque growth (the brighter indentation seen below the T4 wild-type plaque). This suggests that phage T4-infected bacteria can exist well beyond the visible boundaries of their plaques, supporting a diffusion-based alternative to the “swimming” proposal discussed in Section 6. This figure is based on the fifth figure presented in Abedon [10], where additional colliding-plaque images are presented. See in that same publication additional evidence that the area of influence of wild-type phage T4 plaques extends well beyond their visible centers. This figure is reproduced under a Creative Commons Attribution (CC BY) license.

While this phenomenon is visually interesting, it has been recognized for decades that phage mutants appearing on plaque peripheries may acquire rapid lysis-like phenotypes, contrasting with the slower lysis associated with lysis inhibition. The parental phages giving rise to these rapid lysis mutants are themselves mutated phages, known as “star mutants” [18,19,20,21] (see Figure 2 for the plaque of a phage T4 rapid lysis mutant, *r48*). This though does not rule out the phenomenon from occurring with wild-type phages. Furthermore, lysis-inhibited plaques can be substantially larger than they appear visually [10] (Figure 2 and Section 6). If such “invisible” phage spreading is also true for phage 0524phi7-1, then this could allow for the spontaneous formation of such mutants outside of a plaque’s visible boundary.

Critical questions that warrant investigation therefore include (i) whether phage 0524phi7-1 exhibits lysis inhibition, as might be expected for a myovirus [24,25,26] (which phage 0524phi7-1 is); (ii) whether satellite plaques represent mutants relative to the original plaque-founding virion; (iii) whether the visibly unlysed lawns immediately surrounding visible 0524phi7-1 plaques contain still intact, lysis-inhibited, phage-infected bacteria.

## 5. “Multi-Day Plaque Enlargement”

With regard to the authors’ observations of plaque growth over long time spans, it is important to recognize that this phenomenon has been seen with other phages. The podovirus T7, for example, classically produces very large plaques [22], which under certain conditions appears capable of continued enlargement over greatly extended periods [23] (Figure 2). However, multiple alternative explanations may exist for this observation of multi-day plaque enlargement by phage 0524phi7-1. One possibility is that it too could be a consequence of the above-noted lysis inhibition collapse phenomenon. That is, plaques increasing in size not visibly but still in terms of phage-infected bacteria (Section 6), that then subsequently lyse. This gives the impression of multi-day enlargement whereas what actually may be going on is multi-day lysis rather than ongoing infection. This would be an alternative to virions continuing to successfully infect at the perimeter of plaques over very long time frames. In the remainder of this section, I explore an additional potential explanation based on a further observation by Rojero et al. [1], that of their excised plaque assay. I then combine these explanations also with that of the clearing of semi-turbid plaques (Section 3) and the formation of satellite plaques (Section 4).

### 5.1. Excised Plaque Assay

Notwithstanding the possibility of lysis inhibition involvement, the finding of propagation from an excised plaque still embedded in 0.4% agarose gel is a fascinating result, one that is potentially consistent with the noted multi-day plaque enlargement. A possible issue with that experiment, however, is the carryover of nutrients and other materials from the agarose gel, which could have the effect of restoring the phage infectivity of the day-old lawn upon which they were placed. An equivalent result nonetheless was not seen with the application of sucrose gradient-purified virions that had been diluted in broth, suggesting that the carryover of nutrients specifically does not underlie this result.

A potentially better control therefore could have been to place those purified virions in virgin media-containing solidified 0.4% agarose gel for application to the mature lawn (thereby assessing the role of agarose), or alternatively to apply more than 100 phages with or without the agarose (thereby measuring the impact of higher phage multiplicities as likely would have been plaque associated). Additional alternative experiments would involve placing purified phages within media that have been spent via bacterial growth to increasing extents or instead to employ phage-lysed media. These experiments are to determine the impact of bacterial metabolites and phage lysis products on the Rojero et al. [1] excised plaque result.

### 5.2. Impact of Lysis-Released Endolysin?

Endolysins are cell wall-degrading enzymes [27]. Phages use these as part of the mechanism they employ to lyse bacteria at the end of phage latent periods. Endolysins also are released from bacteria as functional enzymes upon this lysis [28]. Especially as the *B. thuringiensis* phage host is Gram-positive, it is conceivable that what was carried over to the plate within the excised agarose was lysis-released phage-encoded endolysin. This endolysin could potentially lyse otherwise recalcitrant mature bacterial cells independent of the action of carried-over phage virions.

Though examples of endolysin activity against physiologically older Gram-positive bacteria do not appear to be common, Gutiérrez et al. [29] demonstrated substantial activity by the endolysin LysH5 against 24 h *Staphylococcus aureus* along with *Staphylococcus epidermidis* biofilms, reducing bacterial numbers by up to multiple logs upon 6 h treatment. Alreja et al. [30] treated 24 h *Streptococcus pneumoniae* biofilms with Cpl-1 and SP-CHAP endolysins, reducing numbers of bacteria by about 1 log_10_ with a 1 h treatment. Liu et al. [31] treated both 24 and 72 h *Staphylococcus aureus* biofilms with the endolysin, LysSYL. The 24 h biofilms could be reduced by 1 log_10_ bacteria in 1 h. The 72 h biofilms were mostly less susceptible to LysSYL even after 5 h treatments, but nonetheless LysSYL still showed significant anti-biofilm activity. Though not perfectly analogous systems, this existence of some endolysin activity against 24 h or older biofilms suggests that it is at least plausible that a phage 0524phi7-1-encoded endolysin could be active also against day-old lawn bacteria.

Endolysin-mediated lysis and the associated removal of bacteria—described as “lysis from without” (though not to be confused with whole-virion mediated lysis from without [32])—could possibly then modify the metabolism of remaining bacteria, making them again susceptible to infection by the virions found in the excised plaque. It is similarly possible that endolysin release over the course of regular plaque growth contributes to the observed multi-day plaque enlargement, whether entirely due to endolysin action or in combination with ongoing phage infection to cause the extended plaque growth.

### 5.3. The Spot-on-Lawn Assay

Evidence for a possible endolysin role in lawn clearing comes as well from what is described as the plate lysis assay [33,34], also dubbed the spot-on-lawn assay [35]. This is a method in which endolysin-containing fluids are spotted onto bacterial lawns usually in 10 μL drops, with lawn clearing under those drops representing a positive lysis from without of bacteria result. Lawns at the time of endolysin application tend to be fresh, however, rather than having been allowed to first mature. Thus, this assay is not directly applicable to the placing of plaque-associated agarose onto a day-old bacterial lawn.

Nevertheless, the greater the endolysin concentration applied, then the greater the width of the resulting clearing (or lysis zone or spot) [35,36]. This implies an endolysin ability to diffuse within agar-based media beyond the point of application as well as have an impact as surrounding lawns mature. Substantial spread can also be seen away from bacteria that are expressing and then releasing cloned endolysin in the presence of susceptible bacterial lawns [34]. In some cases, the lysis becomes less complete in the periphery of these spots [36], presumably due to a combination of lawns maturing and endolysin concentrations declining during their spread. In other cases, however, the spots appear to remain fully clear in their peripheries [34,35]. Rodríguez-Rubio et al. [37] meanwhile demonstrated endolysin (λSa2lys) mediated lysis of broth cultures of stationary phase *Streptococcus agalactiae* that was nearly as rapid as lysis of log-phase bacteria.

Thus, it seems plausible that endolysin released in the course of application of plaque materials could either be completely responsible for the lawn clearing observed by Rojero et al. [1] in their excised plaque assay, or instead could be facilitating phage infection—with either or both leading to the observed mature lawn clearing. The purified phages suspended in fresh media that were applied as a phage- and media-only control, by contrast, would lack an endolysin component. To the extent that phage infections are at least somewhat responsible for the clearings found under excised plaques, then the conclusion that “the infectivity of phage 0524phi7-1 depended on its history” may still be correct, with those whole phages potentially also contributing to multi-day plaque enlargement. That is, carried-over lysin could facilitate phage infection success, though this possibility needs to be tested.

### 5.4. An Important Role of Phage 0524phi7-1 Endolysin?

It is possible that plaque-associated endolysin alone is responsible for both lawn clearing by an excised plaque and subsequent plaque (as a lysis zone) enlargement. Experimentally, especially the first possibility could be explored by first removing virions from a phage lysate [28] and then applying the remaining media to a day-old lawn as a drop to see if the same clearing is observed without—as well as with—the presence of whole phages. The bottom line nonetheless is that more experiments need to be performed to confirm explicitly that phage 0524phi7-1 is capable of producing true phage T7-like multi-day plaque enlargement without substantial endolysin-mediated lysis from without.

The existence of satellite plaques (Section 4) in combination with the lysis inhibition hypothesis presented at the start of this section (Section 5), as well as the endolysin discussion found immediately above, suggests the following scenario for multi-day plaque enlargement: (i) Early visible phage 0524phi7-1 plaque formation occurs via a standard lytic infection mechanism of plaque enlargement [38]. (ii) As the bacterial lawn matures, phage replication especially on the surfaces of growing lawn bacterial microcolonies occurs [39]. The associated lawns remain turbid, however, due to substantial display of lysis inhibition (Section 4 as well as Section 6) in combination with delays in phage penetration fully into lawn microcolonies (Section 3). (iii) Over time, endolysin released from lysing bacteria lyses both still phage-infected bacteria and any still-uninfected bacteria, giving an appearance of a continually growing plaque, starting with the initially turbid plaque peripheries (Section 3). (iv) Prior to that endolysin-mediated lysis from without, satellite plaques appear, as explained in Section 4. The entire process of plaque enlargement in any case is driven by diffusion, both of free endolysin and virions, with the latter addressed in Section 6.

## 6. Alternative to Swimming Phages

The suggestion of virion spread outside of a plaque’s visual perimeter (Section 4) may largely correspond in its apparent consequences to this statement by Rojero et al. [1]: “Phages migrated away from the central, clear plaque region, without lysing cells while migrating.” That spread, however, is readily explainable by mechanisms other than “the swimming of phage 0524phi7-1” suggested by Rojero et al. Instead, this could be due simply to virion diffusion through volumes not yet occupied by lawn microcolonies [40]. This is in addition to the initial phages that come to adsorb microcolonies not displaying lysis inhibition.

These leading-edge phages during plaque formation should be able to speedily contribute to ongoing but otherwise invisible virion spread, contrary to the suggestion of migration without lysis, as at least some phage-induced bacterial lysis would occur. This process thus could represent the normal reaction–diffusion (i.e., infection–diffusion) mechanism of plaque spread [41,42]. This though is with reduced phage-induced lysis due to lysis inhibition occurring behind the reaction–diffusion leading edge, where free phages should be found in greater numbers [17]. This delay in lawn lysis may be enhanced by lawn microcolonies enlarging with time, making them less readily penetrated by phage virions (Section 3), and by lawn bacteria also entering a lysis-recalcitrant stationary phase [43].

That is, lysis inhibition is induced by phage secondary adsorption to already phage-infected bacteria. Some of the phages infecting at the leading edge of plaque formation, however, should be able to avoid these secondary adsorptions along with the resulting lysis delay, due to the expected lower virion concentrations found there. This leading edge lack of lysis inhibition is evidenced by phage T4 wild-type, lysis inhibition-displaying plaques, which appear to display an otherwise invisible area of influence in the surrounding bacterial lawn that is approximately as large as that displayed by a lysis inhibition-defective phage T4 mutant (*r48*) (Figure 2 and next paragraph). That is, plaques produced by phages displaying rapid lysis are expected to be substantially larger [41], but in terms of their area of influence they in fact are not substantially larger [10]. This apparent contradiction can be explained by wild-type phages spreading invisibly due to especially plaque leading-edge infections not displaying lysis inhibition.

The larger-than-expected area of influence of wild-type lysis-inhibited plaques was first demonstrated by Hershey and Rottman [44] in 1949 (their second figure), and experimentally repeated as presented in Abedon [10]. Figure 2 illustrates the phenomenon here, showing colliding plaques of wild-type phage T4 and phage T7, where the latter appears to be unable to progress into the area surrounding the visible portion of the phage T4 plaque. That stalling of phage T7’s progression into the bacterial lawn surrounding phage T4 wild-type plaques presumably is because of the bacteria there being already phage T4-infected. Thus, reaction–diffusion followed by lysis inhibition results in areas of phage infection within a bacterial lawn that nevertheless normally are not visible as larger plaques.

It is important to note also that the “swimming” hypothesis would require a novel, active, and likely energy-dependent mechanism for virion movement. In contrast, this alternative reaction–diffusion–delay (RDD) explanation relies on the well-established process of passive virion movement along with the extensively documented lysis inhibition phenomenon. The RDD hypothesis thus represents a more parsimonious interpretation that does not require proposing new biology. Additionally, while Rojero et al. [1] attempt to rule out host-mediated virion transport, their evidence relies on gel percentage data from a different phage system. The exclusion of host-mediated movement based on such indirect evidence, combined with the proposal of an entirely novel “swimming” mechanism, represents insufficient justification for this extraordinary claim. Given also the alternative RDD explanation presented here, direct experimental confirmation is required before the “swimming” hypothesis can be accepted. This includes testing whether lysis inhibition plays a prominent role in phage 0524phi7-1 plaque dynamics and more effectively ruling out host-mediated virion transport.

## 7. Concluding Remarks

It is entirely possible that phage 0524phi7-1 does not exhibit lysis inhibition. However, given the potential relevance of that phenomenon to the observed characteristics, testing for lysis inhibition in this system would be valuable. For example, see the lysis profile experiments of Rajnovic et al. [45] where such lysis inhibition is readily observed, as well as non-T4 systems in which lysis inhibition has also been observed [24,25,26,46,47]. This suggested testing is particularly important as lysis inhibition appears to be an underappreciated behavior in contemporary phage research, despite its historical [24,48] and potential ecological significance [10].

What makes phage 0524phi7-1 particularly intriguing is that it is a myovirus possibly displaying an ever-enlarging plaque phenotype. That contrasts instead with the podovirus T7, where this large plaque behavior has been classically described [22,23]. As noted, however, the phenomenon in phage 0524phi7-1 might represent a manifestation of the lysis inhibition/lysis inhibition collapse phenomenon. In addition, it could be due to a lysis from without impact of free phage-encoded endolysin on these Gram-positive bacterial lawns.
